# Overexpression of Adenosine A_2A_ Receptors in Rats: Effects on Depression, Locomotion, and Anxiety

**DOI:** 10.3389/fpsyt.2014.00067

**Published:** 2014-06-13

**Authors:** Joana E. Coelho, Pedro Alves, Paula M. Canas, Jorge S. Valadas, Tatiana Shmidt, Vânia L. Batalha, Diana G. Ferreira, Joaquim A. Ribeiro, Michael Bader, Rodrigo A. Cunha, Frederico Simões do Couto, Luísa V. Lopes

**Affiliations:** ^1^Faculty of Medicine of Lisbon, Instituto de Medicina Molecular, University of Lisbon, Lisbon, Portugal; ^2^Faculty of Medicine of Lisbon, Institute of Pharmacology and Neurosciences, University of Lisbon, Lisbon, Portugal; ^3^CNC-Center for Neurosciences and Cell Biology, University of Coimbra, Coimbra, Portugal; ^4^Faculty of Medicine, University of Coimbra, Coimbra, Portugal; ^5^Max-Delbrück-Center for Molecular Medicine (MDC), Berlin, Germany

**Keywords:** adenosine A_2A_ receptors, memory, anxiety, depression, stress, locomotion, dopamine

## Abstract

Adenosine A_2A_ receptors (A_2A_R) are a sub-type of receptors enriched in basal ganglia, activated by the neuromodulator adenosine, which interact with dopamine D_2_ receptors. Although this reciprocal antagonistic interaction is well-established in motor function, the outcome in dopamine-related behaviors remains uncertain, in particular in depression and anxiety. We have demonstrated an upsurge of A_2A_R associated to aging and chronic stress. Furthermore, Alzheimer’s disease patients present A_2A_R accumulation in cortical areas together with depressive signs. We now tested the impact of overexpressing A_2A_R in forebrain neurons on dopamine-related behavior, namely depression. Adult male rats overexpressing human A_2A_R under the control of CaMKII promoter [Tg(CaMKII-hA2AR)] and aged-matched wild-types (WT) of the same strain (Sprague-Dawley) were studied. The forced swimming test (FST), sucrose preference test (SPT), and the open-field test (OFT) were performed to evaluate behavioral despair, anhedonia, locomotion, and anxiety. Tg(CaMKII-hA2AR) animals spent more time floating and less time swimming in the FST and presented a decreased sucrose preference at 48 h in the SPT. They also covered higher distances in the OFT and spent more time in the central zone than the WT. The results indicate that Tg(CaMKII-hA2AR) rats exhibit depressive-like behavior, hyperlocomotion, and altered exploratory behavior. This A_2A_R overexpression may explain the depressive signs found in aging, chronic stress, and Alzheimer’s disease.

## Introduction

Adenosine is a purine nucleoside, which acts as neuromodulator in several brain areas, playing important fine tuning influences on other neurotransmitters (1). It has an important role in central nervous system and its involvement in a wide range of brain processes and diseases has been researched, namely sleep (2, 3), epilepsy (4, 5), panic disorder (6), anxiety (7), Alzheimer’s disease (8), Parkinson’s disease (9), and schizophrenia (10).

So far, four adenosine receptors have been cloned and characterized: A_1_R, adenosine A_2A_ receptor (A_2A_R), A_2B_R, and A_3_R. A_1_R and A_3_R are coupled to Gi proteins, inhibiting cAMP production; A_2A_R is coupled to Gs proteins, stimulating cAMP production; A_2B_R is coupled to Gs and to Gq proteins, stimulating cAMP production, and phosphatidylinositol signal pathway activation, respectively (11). These receptors are not uniformly distributed in the central nervous system. A_1_R is highly expressed in brain cortex, cerebellum, hippocampus, and dorsal horn of spinal cord (1, 11, 12). A_2A_R is highly expressed in the olfactory bulb and in the GABAergic neurons of caudate–putamen, nucleus accumbens, and tuberculum olfactorium (1, 11). A_2B_R and A_3_R are also present in the brain, however, in low levels (11).

Adenosine A_2A_ receptor activation influences the function of several receptors, but the interaction with dopamine D_2_ receptor (D_2_R) is one the most intensively studied (1). Dopamine is a catecholamine neurotransmitter. It activates five known types of receptors, D_1_R–D_5_R, which may be grouped in D_1_-like receptors – D_1_R and D_5_R – and D_2_-like receptors – D_2_R, D_3_R, and D_4_R (13). Dopaminergic neurons are mostly localized in the arcuate nucleus of hypothalamus, substantia nigra pars compacta, and ventral tegmental area. From the substantia nigra–ventral tegmental area complex, three distinct dopamine projection pathways are formed (14). In one of them, axons project to cortical areas, particularly to the frontal cortex, forming the classically described mesocortical pathway. In another pathway, classically known as mesolimbic pathway, axons project to the nucleus accumbens, amygdala, and hippocampus. Due to their functional interrelationships, these two pathways are commonly referred as a single system – the mesocorticolimbic system (15). This system is involved in emotional response, motivation, reward, addiction, and learning. Its role has been emphasized in the pathophysiology of schizophrenia, depression, and drug addiction, and it appears also to be implicated in anxiety disorders (16–20). In the third pathway, axons project to the striatum, forming the classically described nigrostriatal pathway. This pathway integrates the neural circuits of the basal ganglia responsible for motor control and its malfunctioning is classically involved in the pathophysiology of Parkinson’s disease (21), although recent evidence also points to a very important role in the motor changes associated with severe depression (22).

Adenosine A_2A_ receptor and D_2_R are co-localized in the dorsal and ventral striatum and are reciprocal inhibitors: on one hand, A_2A_R–D_2_R heteromers are formed and, when the A_2A_R is activated, conformational changes are transferred to the D_2_R – this leads to a reduction in D_2_R recognition and signaling (23, 24); on the other hand, D_2_R activation inhibits cAMP mediated-effects of A_2A_R by inhibiting adenylyl-cyclase (23, 24).

Knowing these possible interactions between A_2A_R and dopamine, we investigated the impact of A_2A_R overexpression in cortical areas onto dopamine-related behavior. Thus, in the present work a group of behavioral tests was performed in order to analyze the effect of A_2A_R overexpression: (1) sucrose preference test (SPT), considered for a behavioral evaluation of anhedonia (25); (2) forced swim test to evaluate motivation and behavioral despair (26, 27); (3) open-field test (OFT) to study locomotor activity and anxiety-like behavior (28, 29).

## Materials and Methods

### Animals

**Figure 1:**
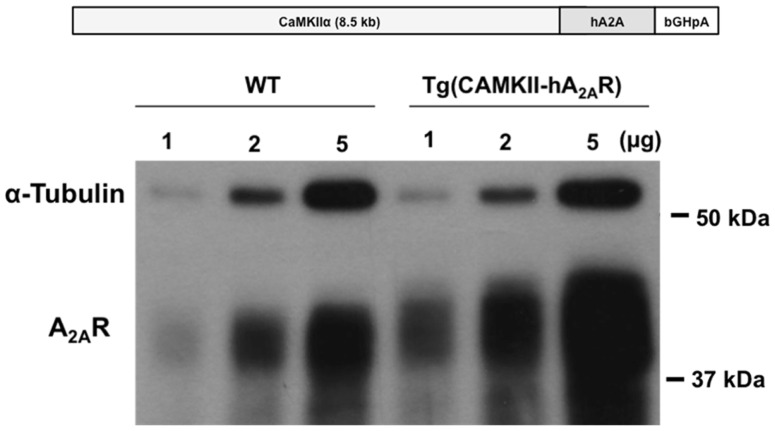
**Neuronal overexpression of adenosine A2A receptor (A_2A_R) in Tg(CaMII-hA2AR) rats**. A_2A_R overexpression in the striatum of Tg(CaMKII-hA2AR) rats compared to WT animals was confirmed by western blotting.

Animal procedures were performed in accordance with the guidelines of the European Community guidelines (Directive 2010/63/EU), Portuguese law on animal care (1005/92), and approved by the *Instituto de Medicina Molecular* Internal Committee and the Portuguese Animal Ethics Committee (*Direcção Geral de Veterinária*). Transgenic rats with an overexpression of the human A_2A_R under the control of the CaMKII promotor, tg(CaMKII-hA2AR), were generated by microinjection of a linearized DNA construct into the male pronucleus of Sprague-Dawley rat zygotes with established methods (30). The construct contained a full-length human A_2A_ cDNA cloned into an expression vector with the 8.5 kb mouse CaMKIIα promoter (31) and a polyadenylation cassette of bovine growth hormone (see Figure 1, top panel). Sprague-Dawley wild-type (WT) rats were used as controls. *Genotyping*: transgenic rats were identified by PCR (30 cycles, 58°C annealing temperature) of their genomic DNA isolated from ear biopsies by the use of the transgene-specific primers CaMKII-hA2A and rat β-actin primers as an internal control (Invitrogen). According to the performed RNase protection assay, these animals expressed A_2A_R pre-dominantly in the brain. qPCR and Western blotting showed that the overexpression was mostly in hippocampus and cortex; there was also overexpression in striatum, however at a lesser extent (Figure 1). Nine week-old WT and transgenic Sprague-Dawley (CaMKII-hA2AR) male rats were used. They were maintained in groups of three in appropriate cages with food and water *ad libitum*, temperature of 21 ± 0.5°C, humidity of 60 ± 10%, and 12 h light/dark cycles beginning at 8 a.m.

### Behavioral testing

The behavioral testing was performed as before (32), during the light period of the cycle, in a silent room, under dim light. From the first to the third day of experiments, the animals were handled for approximately 1 min each. On the fourth day, at 2.30 p.m. the OFT was done. On the fifth day, at 1 p.m. the SPT was initiated. This test was concluded on the seventh day at 9 a.m. On the seventh and eighth day, both at 2 p.m., the forced swim test was executed.

#### Open-field test

The rats were placed in a designated corner of a square apparatus, surrounded by vertical walls (66 cm × 66 cm × 66 cm) – open-field arena. They freely explored the maze for 5 min. Their movements were recorded and analyzed using the video-tracking software – SMART^®^. The reference point used by the software to determine the position of the animal was the center of the rat’s dorsum (also true for the other experiments). Three different zones were defined for analysis (29): (1) the area adjacent to the wall (1896 cm^2^); (2) the central area of the arena (552 cm^2^); (3) the intermediary area between the two previous ones (1908 cm^2^). The percentage of time spent in each zone, the total distance traveled, the average speed (calculated after the elimination of the resting time), and the number of rearings and defecations were determined. At the end of the 5 min test, the rat was removed from the open-field arena and placed into its home cage.

#### Elevated plus maze

The maze is shaped like a plus sign and consists of two “open” (no walls, 5 cm × 29 cm) and two “closed” 122 (5 cm × 29 cm × 15 cm) arms, arranged perpendicularly, and elevated 50 cm above the floor. Each animal was placed on the center of the equipment, facing an open arm. Each test lasted 5 min and all testing sessions were performed between 10:00 a.m. and 17:00 p.m. in a sound attenuated room. The maze was cleaned with a 70% ethanol solution between each animal. The total time spent in the open arms and the total arms entries (number of entries in open + closed arms) were used as anxiety and locomotor parameters as before (32).

#### Sucrose preference test

Rats were given two previously weighed bottles: with 1% (w/v) sucrose solution (33). The bottles had the same characteristics and approximately the same volume of liquid and were positioned side-by-side at the rear of the cage. The rats had free access to both bottles. The position of the bottles in the cage is switched halfway through this period. There was no food or water deprivation before the test. The bottles were weighed again at 48 h and the consumed weight of each liquid was determined. The sucrose preference was calculated according to Bekris et al. (34):

$$\text{SP}=\frac{\text{sucrose solution intake}\;\left(\text{g}\right)}{\text{sucrose solution intake}\;\left(\text{g}\right)+\text{water intake}\;\left(\text{g}\right)}\times 100.$$

#### Forced swim test

On the first of the two test days, all animals were gently placed individually in a vertical Plexiglas cylinder (height: 45 cm, diameter: 19 cm) filled with 23°C tap water at a depth that made it impossible for rats to reach the bottom with hind paws (28–30 cm). The animals were removed from the water after 10 min, and dried before being returned to their home cages. The water was changed after each session. On the next day, the procedure was repeated with two differences: the animals were removed from the water after 5 min, instead of 10 min; the session was video-recorded. An observer blinded to the animal group analyzed the videos. Three different behaviors were considered: (1) immobility – according to the criteria of Porsolt et al. (35), a rat is judged to be immobile when it floated passively, making only small movements to keep its nose above the surface; (2) climbing (or thrashing) – upward-directed movements with its forepaws, in and out of the water, along the side of the swim chamber; (3) swimming – active movements (usually horizontal) more than necessary to merely maintain its head above the water (36, 37). Diving and face shaking behaviors were not considered.

The time (*t*) spent in immobility and climbing was measured; the time spent swimming was calculated: *t* swimming = 5 − (*t* climbing + *t* immobility). Additionally, the latency to the first bout of immobility was determined (38): period of time since the beginning of the rat mobilization in the water until the first episode (at least 1 s) of immobility.

#### Western blotting

The animals were killed by decapitation after anesthesia under halothane atmosphere. After decapitation the brain was rapidly removed and the striata were dissected rapidly frozen in liquid nitrogen for further analysis. Samples were denatured by heating at 70°C for 30 min for A2AR. Samples and molecular weight marker were resolved by SDS-PAGE (8 or 10% for resolving and a 5% for stacking gels) in denaturing conditions and electro-transferred to PVDF membranes (Millipore). Membranes were blocked with 5% non-fat dry milk in TBS-T (Tris buffer saline with 0.1% Tween-20, 200 nM Tris, 1.5 M NaCl). After washing with TBS-T, membranes were incubated with primary antibody in TBS-T with 3% BSA. Secondary antibody incubation was in 5% non-fat dry milk in TBS-T. Primary antibody was mouse anti-A2AR (1:2000, Upstate/Millipore – 05-717, Darmstadt, Germany), secondary antibodies conjugated with horseradish peroxidase were goat anti-mouse (Santa Cruz Biotechnology, Heidelberg, Germany). Chemoluminescent detection was performed with ECL-PLUS western blotting detection reagent (GE Healthcare) using X-Ray films (Fujifilm).

### Statistical analysis

The software used to perform the statistical analysis was Prism 5 – GraphPad software^®^. Unpaired *t* test with Welch’s correction was applied to compare the differences between groups. *p* < 0.05 was considered as statistical significant. Data are expressed as means ± SEM.

## Results

### Weight

**Figure 2:**
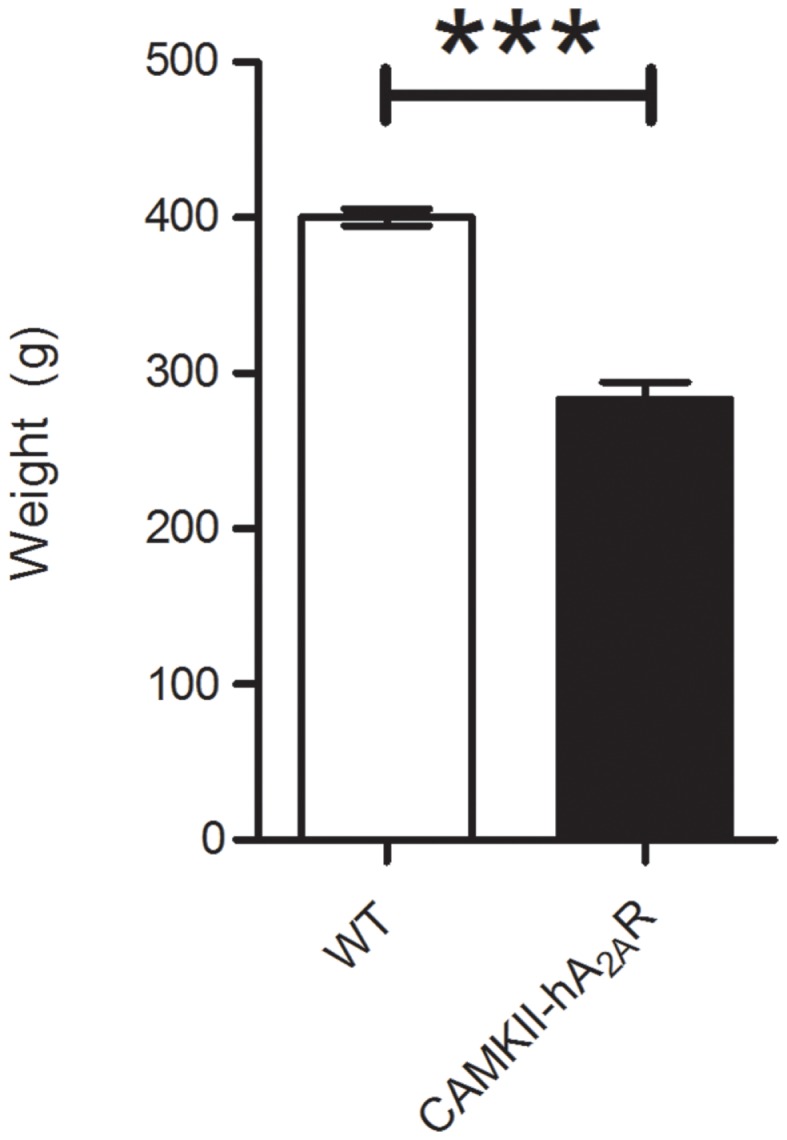
**Weight control of Tg(CaMKII-hA2AR) animals**. Results are expressed as mean ± SEM. ***p < 0.001 WT: *n* = 12; Tg(CaMKII-hA2AR): *n* = 4.

The weight of transgenic CaMKII-hA_2A_R rats was significantly lower than WT rats (283 ± 11 vs. 400 ± 5 g; *p* < 0.001) (Figure 2).

### Open-field test

**Figure 3:**
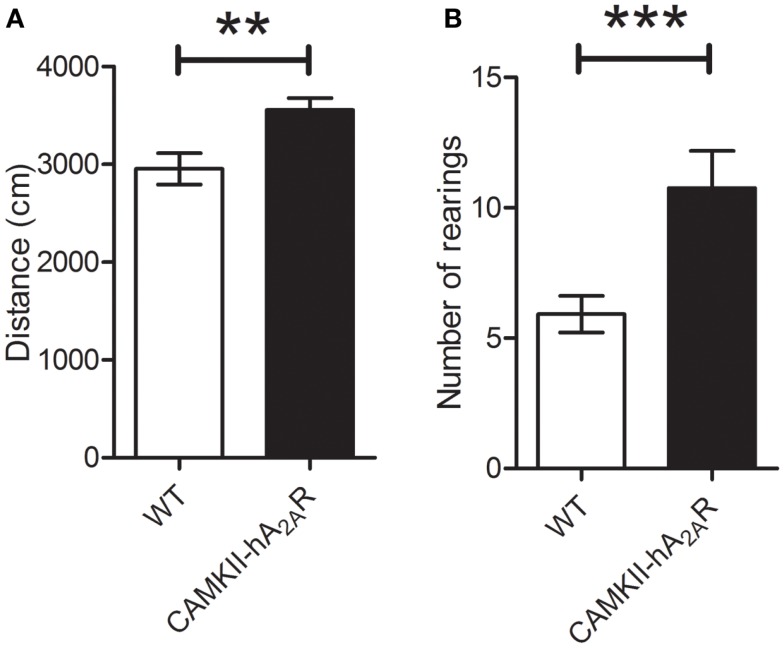
**Open-field test**. **(A)** Distance covered in centimeter. **(B)** Number of rearings – results are expressed as mean ± SEM. **p < 0.01; ***p < 0.001. WT: *n* = 12; Tg(CAMKII-hA2AR):*n* = 4.

The total distance covered in the open-field arena was significantly higher in Tg(CaMKII-hA2AR) rats [WT: 2956 ± 160 cm; Tg(CaMKII-hA2AR): 3644 ± 64 cm; *p* = 0.0013], and was accompanied by a significant increase in the number of rearings [WT: 5.9 ± 0.7; Tg(CaMKII-hA2AR): 10.8 ± 1.4; *p* = 0.0390], suggesting that Tg(CaMKII-hA2AR) rats display hyperlocomotion, Figure 3.

**Figure 4:**
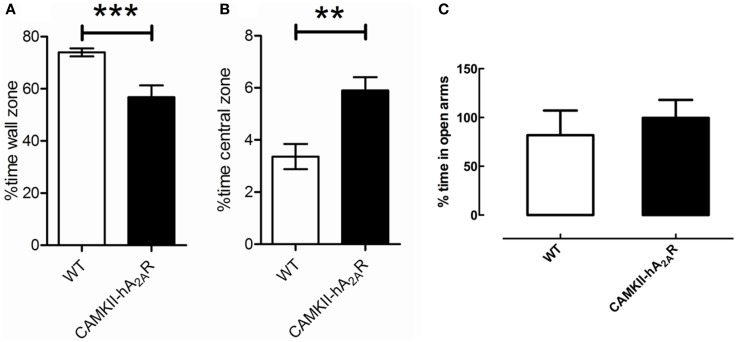
**Open-field test**. Percentage of time spent **(A)** at the wall zone and **(B)** in the central zone. **(C)** Percentage time in the open arms of the elevated plus maze (EPM). Results are expressed as mean ± SEM. **p < 0.01; ***p < 0.001. WT: *n* = 12; Tg(CaMKII-hA2AR): *n* = 4.

Transgenic (CaMKII-hA2AR) rats spent less time at the wall zone [WT: 73.97 ± 1.6%; Tg(CaMKII-hA2AR): 61.59 ± 1.94%; *p* < 0.0001] and more time in the central zone of the open-field box [WT: 3.36 ± 0.48%; Tg(CaMKII-hA2AR): 5.21 ± 0.40%; *p* = 0.0083, Figure 4] suggesting that Tg(CaMKII-hA2AR) rats have increased exploratory behavior. We could not detect significant changes in the anxious behavior evaluated by EPM (Figure 4C). There were no statistical significant differences regarding the number of defecations and the average speed between the two groups (data not shown).

### Sucrose preference at 48 h

**Figure 5:**
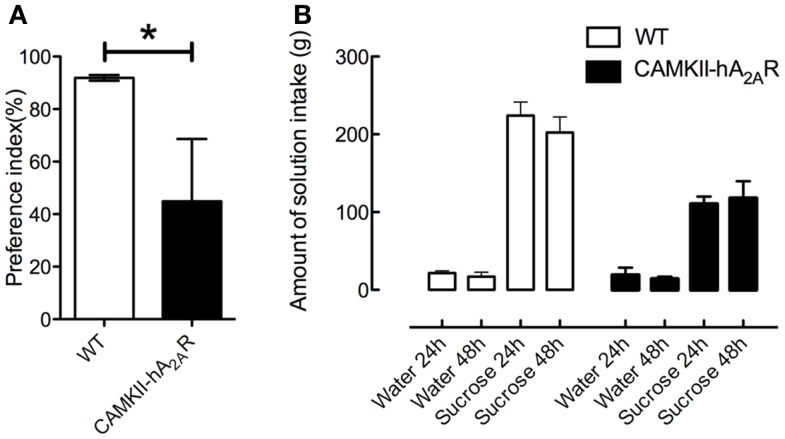
**Sucrose preference test**. **(A)** Preference index at 48 h and **(B)** control of amount of solution intake. Results are expressed as mean ± SEM. *p* < 0.05 WT: *n* = 8; Tg(CaMKII-hA2AR):*n* = 4.

At 48 h, the preference index for sucrose was significantly higher in WT than in Tg(CaMKII-hA2AR) rats [WT: 91.88 ± 1.4%; Tg(CaMKII-hA2AR): 44.85 ± 23.78%, *p* = 0.0081, Figure 5], suggesting that Tg(CaMKII-hA2AR) rats have an anhedonic-like phenotype.

### Forced swim test

**Figure 6:**
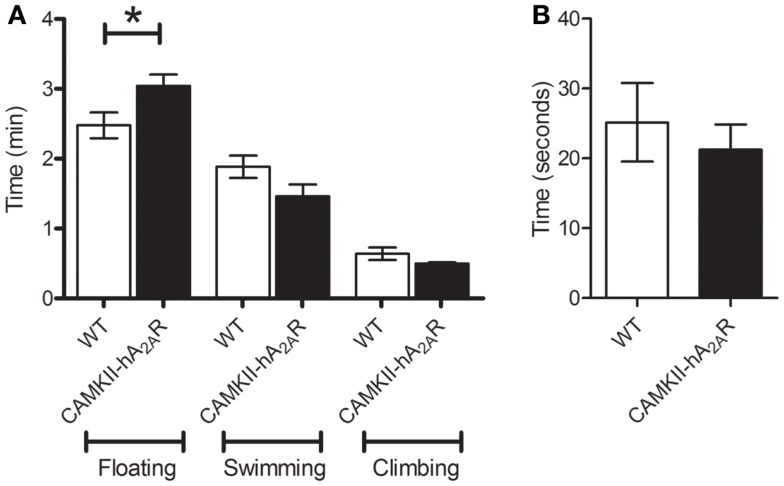
**Forced swim test**. **(A)** Time spent floating, swimming, and climbing; **(B)** latency to the first period of immobility – results are expressed as means ± SEM. *p* < 0.05 WT: *n* = 12; Tg(CaMKII-hA2AR):*n* = 4.

Transgenic (CaMKII-hA2AR) rats spent significantly more time floating than WT rats (2.47 ± 0.18 vs. 3.04 ± 0.16 min, *p* = 0.0452, Figure 6A) indicating that transgenic animals have increased behavioral despair. No significant changes were apparent in both swimming and climbing times, despite a tendency to lower performance in Tg(CaMKII-hA2AR) animals (Climbing: 0.63 ± 0.08 vs. 0.50 ± 0.02 min, *p* = 0.1486; swimming: 1.88 ± 0.16 vs. 1.46 ± 0.17 min, *p* = 0.1060).

The latency to the first period of immobility was not different between WT and transgenic animals [WT: 25.2 ± 5.6 s; Tg(CaMKII-hA2AR): 26.6 ± 3.8 s; *p* = 0.8304, Figure 6B].

## Discussion

We now report that rats overexpressing A_2A_R in the hippocampus, cortex and striatum, display depressive-like behavior, increased locomotor activity, and altered exploratory behavior.

### Depressive-like behavior

The SPT is considered a behavioral test for anhedonia, defined as the inability to feel pleasure from usually enjoyable activities, a core symptom of depression. Decreased sucrose solution intake, resulting from chronic mild stress, is used as a model of depression in rats, and can reversed by the administration of antidepressants (25, 39, 40). Thus, a decreased sucrose preference is associated to depressive-like behavior, specifically anhedonia. In our study, rats overexpressing A_2A_R, had a decrease in the preference index for sucrose solution at 48 h.

Additionally, when rats are placed in an inescapable cylinder of water – forced swim test – following initial escape-directed movements, they develop an immobile posture (27, 36). Immobility indicates either a failure in the persistence to escape (behavioral despair) or the act of giving up an active form of coping with the stressful stimuli (36). The immobility period and the latency to the first bout of immobility both decrease with the administration of antidepressants (38, 41, 42). Thus, an increased period of immobility and reduced latency to immobility represent depressive-like behavior. A_2A_R overexpressing animals spent more time floating, while no significant differences were observed in the time spent swimming, climbing, or latency to immobility. These behaviors, again, suggest a depressive-like phenotype.

Genetic inactivation and pharmacological blockade of A_2A_R have antidepressant-like effects (43). However, selective A_2A_R agonists are also able to decrease the immobility time in the forced swim test (44, 45). Our observations indicate that A_2A_R overexpression in neurons results in a depressive-like phenotype. Interestingly, depressive symptoms are found in Alzheimer’s disease patients, which have an abnormal accumulation of A_2A_R in cortical areas (8).

### Locomotor activity

The increase in locomotor activity displayed by the Tg(CaMKII-hA2AR) rats is in accordance with the hypolocomotor phenotype of mice with genetic deletion of A_2A_R (46, 47). However, this genetic manipulation does not reflect the effect of acutely administered A_2A_R agonists that reduce locomotor activity (48, 49). The A_2A_R–D_2_R interaction hypothesis of reciprocal inhibition is not suitable to explain the obtained result: with increased amounts of A_2A_R, we would expect a decreased activation of D_2_R, which results in hypo-locomotion; and it is known that D_2_R antagonists suppress locomotion (50, 51).

### Anxious-like behavior

Since rat is a gregarious animal, which usually lives in small spaces, its separation from its social group and placing in a large arena trigger an anxious behavior. In these situations, they naturally display a propensity to walk close to the walls and to avoid open spaces, a behavior called thigmotaxis. Based on this, it is considered that increased time spent on the central zone of the OFT represents a less anxious behavior (29, 52). Similarly, rats display a pre-disposition toward protected, enclosed areas, which is in conflict with their innate motivation to explore new environments. The Tg(CaMKII-hA2AR)rats spent more time on the central zone and less time on the wall zone of the OFT. This increase in exploratory behavior is not a consequence of an anxious-like phenotype of rats overexpressing A_2A_R and is in line is in line with previous studies of the other known model of rats overexpressing A_2A_R receptors in which no difference was found concerning anxiety-like behavior (53). This could also be due to the postulated differences in the cellular origin of A_2A_R, which has been highlighted recently in a study showing that inactivation of striatal A_2A_R s facilitates Pavlovian fear conditioning, whereas inactivation of extrastriatal A2ARs in the forebrain inhibits fear conditioning and also affects anxiety-related behavior (54). Also, there is no evidence for anxiogenic or anxiolytic effects of A_2A_R agonists or antagonists (49, 55).

The A_2A_R–D_2_R interaction hypothesis of reciprocal inhibition does not again provide an explanation for our findings: D_2_R agonists have anxiolytic properties, which are blocked by D_2_R antagonists (56, 57); consequently, we would expect an increased anxious state in animals overexpressing A_2A_R. Similar results obtained with animals overexpressing A_2A_R but controlled by the widespread neuronal promoter enolase (NSE) (53), seem to indicate that the striatal overexpression is not as disturbing as one cortical and hippocampal, dominant in these CaMKII A_2A_ rats.

There could be a mutual influence between anxiety and locomotor activity behaviors. On one hand, the anxiety state can change locomotor activity (58). On the other hand, the anxiety-related tests depend on motor activity (59). However, there is evidence that locomotion and anxiety are differentially regulated by adenosine A_2A_R: studies using A_2A_R knockout mice showed that the hypo-locomotion pattern was equal in homozygous and heterozygous mice, as well as in forebrain selective vs. striatal KO, irrespectively of the effects on anxiety (54, 60).

### Additional and integrative explanations for the observed phenotype

As previously mentioned, the A_2A_R–D_2_R interaction hypothesis of reciprocal inhibition is not enough to explain the hyperlocomotor and the less anxious-like phenotype of Tg(CaMKII-hA2AR) rats. These findings can be explained by two possibilities: (a) these behaviors are regulated by other neurotransmitters influenced by A_2A_R, whose actions are pre-dominant over dopamine action; (b) there are alternative interactions between A_2A_R and dopamine receptors, as some studies about rewarding and habit formation have suggested (46, 61–63). A_2A_R interacts with several G protein-coupled (besides D_2_R), ionotropic, and receptors for neurotrophic factors (1). In this group of interactions, there are some, which can explain the hyperlocomotor and/or the decreased anxious-like behaviors: CB1 (56); delta-opioid (56, 64, 65); NMDA (66, 67); nAch (56, 68); GDNF (69), BDNF (56), or GABA_A_ (70, 71) receptors. Additionally, A_2A_R receptors interact either synergistically or antagonistically with A_1_R receptors (72–74), which, *per se*, influence several other receptors, increasing the complexity of the above mentioned neuromodulation. Furthermore, A_2A_R are also located at glutamatergic synapses (75) and in astrocytes (76), allowing A_2A_R to directly control synaptic transmission and plasticity (77), which we have shown before to be directly involved in adaptive brain behaviors, namely to early-life stress (32). One hypothesis than cannot be ruled out is the possibility that these animals may have adaptive dopamine receptor alterations, which still needs to be determined.

In what concerns to the second hypothesis, two possibilities can be taken into consideration: (1) a single alternative interaction between A_2A_R and dopamine receptors is suitable to explain all behavioral results; (2) different interactions occur in distinct neuronal populations, which control different behavioral processes. In what regards the first possibility, just one of alternative interactions is able to explain all behavioral results – antagonistic interaction between A_2A_R and D_1_R occurring at a network level, considering their scarce co-expression, (23, 61, 78). D_1_R antagonists increase locomotion when administered chronically (51), whereas D_1_R agonists have anxiogenic and antidepressive-like effects (56, 79–81). Therefore, with the A_2A_R overexpression and the associated decrease in D_1_R action, there would be a hyperlocomotor, less anxious, and more depressive-like behavior – which perfectly matches the results we obtained.

If we consider that different interactions control different processes, then, three alternative interactions can explain the increased locomotion and the decreased anxious-like behaviors – particular synergism between A_2A_R and D_2_R mediated by G protein beta/gamma dimers (61, 82), a supposed functional hyperdopaminergic state in Tg(CaMKII-hA2AR) rats (61, 83); and a presumed increased level of DARPP-32 (a downstream effector molecule of D2-like receptors) in CaMKII-hA_2A_R rats (62, 84) – whereas one alternative interaction is able to explain the increased depressive-like behavior – synergism between A_2A_R and mGluR5 (46, 85, 86), which were found to be decreased in the NSE A_2A_ overexpressing rats (53).

## Conclusion

We conclude that Tg(CaMKII-hA2AR) rats overexpressing A_2A_R in hippocampus, cortex, and striatum, have depressive-like behavior and increased locomotor activity. Additionally, we found that the A_2A_R–D_2_R interaction hypothesis of reciprocal inhibition is not sufficient to explain all the observed behavioral outcomes. Finally, we conclude that and A_2A_R overexpression in forebrain is associated with depression, which may explain the depressive signs seen in aging, chronic stress, and Alzheimer’s disease.

## Conflict of Interest Statement

The authors declare that the research was conducted in the absence of any commercial or financial relationships that could be construed as a potential conflict of interest.
